# Enhancement of Intracellular Calcium Ion Mobilization by Moderately but Not Highly Positive Material Surface Charges

**DOI:** 10.3389/fbioe.2020.01016

**Published:** 2020-09-08

**Authors:** Martina Gruening, Sven Neuber, Peter Nestler, Jutta Lehnfeld, Manuela Dubs, Katja Fricke, Matthias Schnabelrauch, Christiane A. Helm, Rainer Müller, Susanne Staehlke, J. Barbara Nebe

**Affiliations:** ^1^Department of Cell Biology, Rostock University Medical Center, Rostock, Germany; ^2^Soft Matter and Biophysics, Institute of Physics, University of Greifswald, Greifswald, Germany; ^3^Colloid and Interface Chemistry, Institute of Physical and Theoretical Chemistry, University of Regensburg, Regensburg, Germany; ^4^Department of Biomaterials, INNOVENT e.V., Jena, Germany; ^5^Leibniz Institute for Plasma Science and Technology e.V. (INP), Greifswald, Germany; ^6^Department Science and Technology of Life, Light and Matter, Faculty of Interdisciplinary, University of Rostock, Rostock, Germany

**Keywords:** human osteoblasts, calcium ion signaling, titanium surface modification, amino polymer, polyelectrolyte multilayer, zeta potential, surface charge, wettability

## Abstract

Electrostatic forces at the cell interface affect the nature of cell adhesion and function; but there is still limited knowledge about the impact of positive or negative surface charges on cell-material interactions in regenerative medicine. Titanium surfaces with a variety of zeta potentials between −90 mV and +50 mV were generated by functionalizing them with amino polymers, extracellular matrix proteins/peptide motifs and polyelectrolyte multilayers. A significant enhancement of intracellular calcium mobilization was achieved on surfaces with a moderately positive (+1 to +10 mV) compared with a negative zeta potential (−90 to −3 mV). Dramatic losses of cell activity (membrane integrity, viability, proliferation, calcium mobilization) were observed on surfaces with a highly positive zeta potential (+50 mV). This systematic study indicates that cells do not prefer positive charges in general, merely moderately positive ones. The cell behavior of MG-63s could be correlated with the materials’ zeta potential; but not with water contact angle or surface free energy. Our findings present new insights and provide an essential knowledge for future applications in dental and orthopedic surgery.

## Introduction

Bone defects due to fractures, infections or tumor resections are one of the main causes of disability in an aging society, leading to a loss of quality of life (Akter and Ibanez, 2016). In orthopedic applications, titanium (Ti) has been and still is the material of choice due to properties such as high strength and corrosion resistance (Hanawa, 2019). To improve the bioactivity of Ti-based materials, several strategies are used, including physical treatments to modify the topography (Wennerberg and Albrektsson, 2009; Nikkhah et al., 2012; Kumar et al., 2019) or chemical treatments to modify the bioactivity (Kokubo and Yamaguchi, 2015; Muderrisoglu et al., 2018; Devgan and Sidhu, 2019) all aiming to optimize the interaction with osteoblastic cells.

Surface properties are one of the main factors influencing the cells’ fate by guiding cellular processes at the interface from the very beginning (Felgueiras et al., 2018; Ferrari et al., 2019). As a result, the advanced cell-material interaction is a pivotal step determining the success of osseointegration, ultimately, the success of implants. Initial processes driven by the surface properties involve cellular attachment, adhesion and spreading (von der Mark and Park, 2013), which further affect other cell activities such as proliferation, differentiation (Bacakova et al., 2011), and intracellular signaling (Anselme et al., 2010; Staehlke et al., 2015, 2018). Stimulating cellular behavior at the interface by acting on surface physico-chemical properties, especially roughness, stiffness, wettability, and surface charge via surface functionalization can be an effective way to improve bone regeneration (Ferrari et al., 2019).

Previous research has shown that osteoblasts favor a certain range of roughness, pore size, wettability, specific biomacromolecules or their biomimetic motifs (Chen et al., 2018) and also stiffness (Abalymov et al., 2020). It could be demonstrated that cells prefer moderately hydrophilic surfaces displaying contact angles between 40° and 65° (Bacakova et al., 2011; Rebl et al., 2012; Mörke et al., 2017). However, other studies have shown that this is not always the case; for instance MG-63 osteoblasts exhibited increased cell attachment and spreading on methylated silicon surfaces with decreasing wettability (Padial-Molina et al., 2011). Similarly, Kennedy et al. (2006) reported that MC3T3-E1 cells displayed improved cell proliferation with increased hydrophobicity and the lowest spreading on the most hydrophilic SAM (self-assembled monolayers) surface. Thus, the contact angle is not a good predictor of cell behavior (Gentleman and Gentleman, 2014).

Furthermore, it has been proven that extracellular matrix (ECM) proteins (mainly collagen, fibronectin, laminin and vitronectin) (Müller et al., 2006; Rico et al., 2009; Chen et al., 2018), their RGD sequence (Arg-Gly-Asp) (Mörke et al., 2017) and cytokines [e.g., basic fibroblast growth factor (bFGF)] (Cao et al., 2015) have the capacity to promote cell attachment.

Some explanations have also been suggested for the surface charge and its effect on the cell-material interaction. Surface charges can generate electrical cues necessary to regulate cell function (De Aza et al., 2003; Finke et al., 2007; Fernández-Yagüe et al., 2019). As human osteoblasts are negatively charged (Rebl et al., 2016), positive surface charges significantly influence cell adhesion (Rebl et al., 2010; Dhowre et al., 2015; Mörke et al., 2017), spreading and proliferation (Staehlke et al., 2019), particularly in the early stages of cell responses. The most detailed study on the effect of surface charges to date is that of Metwally and Stachewicz (2019), indicating a great importance in the development of functionally implantable biomaterials. They reviewed that surface charges determine protein adsorption and thus the subsequent cell adhesion process. Referring to Goldenberg and Steinberg (2010) surface charge seems to be also important for the correct protein localization of signaling molecules in the cell membrane; it has been documented that proteins like GTPases of the Ras, Rho, Arf, and Rab protein families target the plasma membrane through electrostatic interactions (Heo et al., 2006).

The measurement of zeta (ζ) potentials is a suitable technique for characterizing the charging behavior at the solid-liquid interface of modified biomaterial surfaces (Ferraris et al., 2018). There are few papers that address materials such as hydrogels (Schulz et al., 2018), polyelectrolyte multilayers (PEM) (Guo et al., 2018) or metals (Ponsonnet et al., 2003; Nebe et al., 2007) exhibiting a positive ζ-potential enhancing cell processes like cell adhesion, spreading, viability and proliferation. And yet present understanding of the mechanism involved in controlling cell activities via surface charge in tissue engineering is still limited.

In order to gain deeper insights into the impact of surface charges on the cell-material interaction, systematic experiments are required. Our previous work has shown that a plasma polymerized nanolayer of allylamine (PPAAm) provides positive charges on an otherwise negatively charged Ti surface that can boost cell behavior (reviewed in Nebe et al., 2019). The question arose whether a positive ζ-potential is generally considered to be a decisive factor for the cellular outcome.

In the present study, we generated a broad range of surface charges to investigate in detail their influence on osteoblastic cell response via intracellular calcium ion (Ca^2+^) mobilization, cell viability and proliferation. For this purpose, we deposited nine different top layers on Ti substrates from the following three categories: (i) amino polymers, (ii) ECM/peptide motifs, and (iii) PEM. We determined the physico-chemical characterization of these surfaces via water contact angles (WCA), surface free energies (SFE), X-ray photoelectron spectroscopy (XPS) and layer thicknesses, and employed ζ-potential measurements to determine surface charges.

We hypothesize that cell physiology can be improved by material surfaces featuring a certain range of positive ζ-potential.

## Materials and Methods

### Functionalization of Titanium Arrays

**FIGURE 1:**
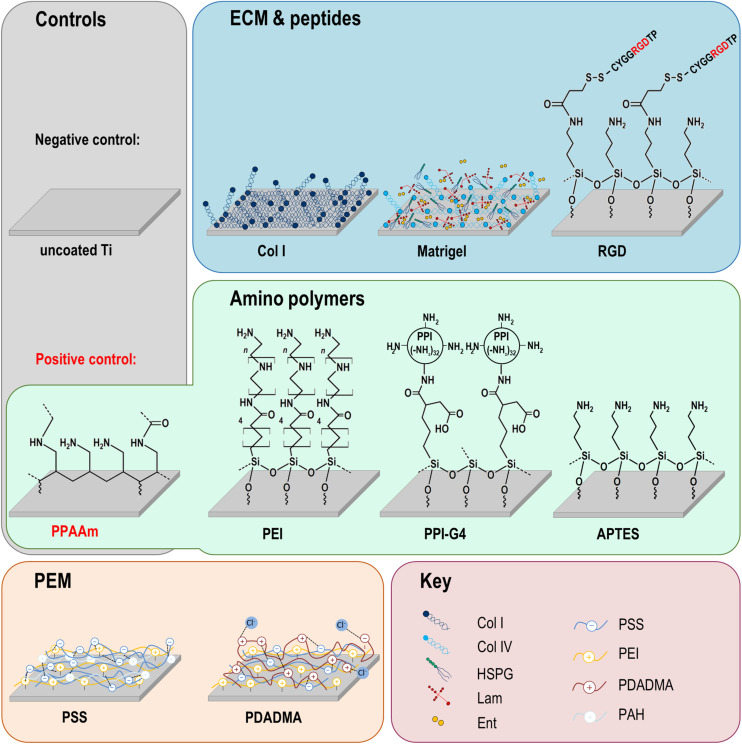
Chemical surface modifications of plane Ti arrays. The modifications can be classified into three groups: amino polymers, extracellular matrix (ECM)/peptide motifs and polyelectrolyte multilayers (PEM). Please note that the molecular structures are schematic formula drawings (created using the freeware ACD/ChemSkretch 2018.2.5, Advanced Chemistry Development, Toronto, ON, Canada), which do not necessarily allow conclusions about the molecular length of the individual layers. Ti, titanium; Col I, collagen-type I; RGD, peptide sequence Arg-Gly-Asp; PPAAm, plasma polymerized allylamine; PEI, poly(ethylene imine); PPI-G4, poly(propylene imine) dendrimer generation 4; APTES, (3-aminopropyl)triethoxysilan; PSS, poly(styrene sulfonate); PDADMA, poly(diallyldimethylammonium chloride); HSPG, heparan sulfate proteoglycans; Lam, laminin; Ent, entactin; Col IV, collagen-type IV; PAH, poly(allylamine hydrochloride).

The following section describes the methods for the diverse Ti surface modifications used for surface charge determination and in the cell biological experiments shown in Figure 1. They are categorized in modifications with amino polymers, ECM/peptide motifs, as well as in PEM. As base material and negative control we used planar silicon arrays sputtered with 100 nm Ti particles obtained from the Center for Microtechnologies (ZFM, University of Technology Chemnitz, Germany) at 1 × 1 × 0.075 cm in size (length × width × depth) for cell analysis, or 2 × 1 × 0.075 cm for ζ-potential analysis as previously reported (Staehlke et al., 2018).

#### Amino Polymers

##### Plasma polymerized allylamine (PPAAm)

As it is known that the positively charged plasma polymerized allylamine (PPAAm) layer can improve several cell functions (Rebl et al., 2012; Mörke et al., 2017; Staehlke et al., 2018; Nebe et al., 2019), this plasma functionalization of Ti was used as a positive control in all experiments. The specimens were coated with the PPAAm nanolayer by using a low-pressure plasma reactor (V55G, Plasma Finish, Germany) (Rebl et al., 2012) with the following parameters: continuous wave oxygen/argon plasma (500 W, 50 Pa, 1000-sccm O_2_, 5 sccm Ar, 60 s), 480 s PPAAm deposition time. PPAAm films have proven to be very robust according to DIN EN 582 (1993), mechanically stable (Fritsche et al., 2009) and can be used for cell experiments for up to 200 days when stored in ambient air (Finke et al., 2014).

##### (3-Aminopropyl)triethoxysilane (APTES)

Ti surfaces were modified with the linker (3-aminopropyl)triethoxysilane (APTES) (Mörke et al., 2017). Prior to modification, the substrates were cleaned and pre-activated by water vapor plasma treatment for 15 s at 0.1 mbar in a microwave. Then, Ti surfaces were coated with 100 mM APTES (abcr, Karlsruhe, Germany) in toluene (Alfa Aesar/Fisher Scientific, Kandel, Germany) for 3 h at 60°C in an incubator (GFL 3032, Burgwedel, Germany) turning at 35 rpm. After carefully washing with ultrapure water, the samples were dried under a stream of pure nitrogen (ultra-high purity 5.0 grade) in a laminar flow box (Herasafe KS 12, Kendro, Langenselbold, Germany). APTES layers are stable up to 1 year when stored under exclusion of light and oxygen.

##### Poly(ethylene imine) (PEI)

Before functionalization, contaminants were removed from the substrate surface by sonicating in acetone (p.a., Sigma-Aldrich Chemie, Taufkirchen, Germany), followed by oxidation of the substrates in a semi-concentrated HNO_3_ solution (38%, 1:1 v/v from concentrated (≥ 65%) nitric acid, obtained from Carl Roth, Karlsruhe, Germany) for 1 week. The wafers were rinsed with ultrapure and sterile water multiple times until a neutral pH was obtained, then dried in a vacuum desiccator (Carl Roth). As described in previous publications, further modification steps were performed under an argon atmosphere in anhydrous solvents (dried with a molecular sieve from Carl Roth) (Eichler et al., 2011; Katzur et al., 2012). The synthesis of carboxylic acid-terminated SAM (SAM-COOH) is based on the protocol by Liu et al. (2002) and was described previously (Katzur et al., 2012). Here, the silanization in a 7-octenyltrichlorosilane solution was carried out for 2 h, the conversion of the terminal alkene groups in a KMnO_4_ solution for 15 min.

For the immobilization of the poly(ethylene imine) (PEI) polymer, the SAM-COOH-modified substrates were first activated via immersion in a solution containing 100 mmol/l *N*-(3-dimethylaminopropyl)-*N’*-ethylcarbodiimide hydrochloride (EDC) and 100 mmol/l *N*-hydroxysuccinimide (NHS) in 0.1 mol/l 2-(N-morpholino) ethanesulfonic acid (sodium salt) (MES) buffer (pH adjusted to 5.4) for 2 h at room temperature (RT). EDC, NHS and MES were purchased from Sigma-Aldrich Chemie of analytical grade. After rinsing once with MES buffer, twice with water and once with methanol (p.a., Fischer Scientific, Schwerte, Germany), the immobilization of the polymer was carried out in a 10 mg/ml PEI solution (linear, Mw = 25 kDa, abcr, Karlsruhe, Germany) in a methanol/water mixture (9:1 v/v) for 1.5 h at RT. Excess polymer was removed by rinsing twice with the methanol/water mixture and once with methanol before the specimens were dried in a desiccator. PEI layers are stable for up to 8 weeks when stored in the dark and under argon atmosphere.

##### Poly(propylene imine) dendrimer (PPI-G4)

In respect to the functionalization with PEI described above, the surfaces were first cleaned by sonication and then oxidized for 1 week. Then a silanization of substrates with 3-(triethoxysilyl)propyl succinic acid anhydride (TESPSA, abcr, Karlsruhe, Germany) was performed according to previously published protocols (Eichler et al., 2011) at elevated temperatures (110°C for 30 min, followed by 130°C for 10 min) under an argon atmosphere. Excess silane was removed by rinsing twice with dry toluene. The specimens were sonicated once with dry dimethyl sulfoxide (DMSO, Fisher Scientific, Schwerte, Germany) and immersed in a 0.5 mmol/l poly(propylene imine) dendrimer generation 4 (PPI-G4, SyMO-Chem, Eindhoven, Netherlands) solution in anhydrous DMSO. The reaction was performed for at least 40 h at RT. Rinsing was performed by sonicating once with DMSO and at least three times with methanol before drying the wafers in a desiccator. PPI-G4 layers are stable for up to 8 weeks when stored in the dark and under argon atmosphere.

#### Extracellular Matrix (ECM) Proteins and Peptide Motifs

##### Collagen type-I (Col I)

Ti substrates were chemically wet-coated with 40 μg/cm^2^ collagen type-I (Col I; rat tail, Corning, Discovery Labware, Bedford, MA, United States) and dried overnight at RT (Rebl et al., 2010). Residues of acetic acid were removed by rinsing twice with sterile ultrapure water. Col I coatings were produced prior to cell experiments and used directly.

##### Basement membrane matrix (Matrigel)

Ti arrays were coated with basement membrane matrix (Matrigel) containing laminin, collagen type-IV, heparan sulfate proteoglycans, entactin and growth factors (Corning, Discovery Labware, Bedford, MA, United States) using a thin-film coating method. For this purpose, Matrigel was thawed on ice and diluted to a concentration of 1 mg/ml using serum-free ice-cold Dulbecco’s Modified Eagle Medium (DMEM, 21063-029, Life Technologies Limited, Paisley, United Kingdom). 200 μl of the solution was added to the Ti substrates (200 μg/cm^2^) with pre-cooled pipette tips and incubated at RT for 1 h under sterile conditions. Unbound material was aspirated and substrates were rinsed gently using serum-free DMEM. Matrigel coatings were produced prior to cell experiments and used directly.

##### Arginine-glycine-aspartic sequence (RGD)

Ti surfaces were modified with a nonapeptide containing the arginine-glycine-aspartic acid (RGD)-tripeptide (complete amino acid sequence C-Y-G-G-R-G-D-T-P, kindly provided by Dr. K. Rischka, Fraunhofer IFAM Institute Bremen, Germany) as reported in Mörke et al. (2017). Prior to peptide coupling Ti substrates was coated with APTES as described above. RGD layers are stable up to 1 year when stored under exclusion of light and oxygen.

#### Polyelectrolyte Multilayers (PEM)

Polyelectrolyte multilayer films were created by dip-coating of oppositely charged polyelectrolytes with a deposition robot (Riegler & Kirstein, Potsdam, Germany) according to Mohamad et al. (2019). Here, we used PEI [branched, Mw = 750 kDa, polydispersity (PDI) = 12.5], polyallylamine hydrochloride (PAH, Mw = 50–60 kDa) and poly(diallyldimethylammonium chloride) (PDADMA, Mw = 322 kDa, PDI = 2.19) as polycations and poly(styrene sulfonate) sodium salt (PSS, Mw = 666 kDa, PDI < 1.2) as polyanion.

PSS-terminated PEM consisted of three double layers with the sequence PEI/PSS/(PAH/PSS)_2_. PDADMA-terminated polyelectrolyte brushes were composed of 10.5 double layers with the sequence PEI/PSS/(PDADMA/PSS)_9_/PDADMA. The layer stability of prepared PEM was verified by AFM measurements [DI Multimode AFM using NanoScope IIIa software from Veeco (Santa Barbara, CA, United States)]. Layers are stable in the range of pH 3–11, in solutions up to 1 M NaCl and after storage in ambient air up to 1 year unchanged.

### Surface Characterization

#### Zeta Potential

ζ-potentials were assessed with the SurPASS^TM^ system (Anton Paar, Ostfildern, Germany) and the associated software Attract 2.1 as previously published (Staehlke et al., 2018). Streaming potentials were measured at pH 6.5–8.0 in a 1 mM KCl solution (VWR International, Darmstadt, Germany) and ζ-potentials at pH 7.4 were calculated via a linear regression using the software GraphPad Prism version 6.05 (*n* = 3, three pairs of samples).

#### Surface Wettability

Surface free energies of the substrate/air interface and WCA were determined by the sessile drop method using the Drop Shape Analyzer—DSA25 (Krüss, Hamburg, Germany) (Staehlke et al., 2018). One μl drops of distilled water and diiodomethane (Sigma-Aldrich Chemie, Taufkirchen, Germany) were deposited on the sample surface (*n* = 3 at 3 drops each). WCA values were calculated with the supplied software (ADVANCE, V.1.7.2.1, Krüss, Hamburg, Germany) via the Young’s equation and the SFE according to Owens, Wendt, Rabel und Kaelble (OWRK).

#### Layer Thickness

Layer thickness was measured with null ellipsometry (Multiskop; Optrel GbR, Sinzing, Germany) as described before (Mörke et al., 2017). Each sample was modeled by 6 slabs in order to account for oxide layers (Si, SiO_2_, Ti, TiO_2_, layer of interest and ambient air). The thickness of the Ti and TiO_2_ layer was determined independently prior to deposition of the layer of interest. Additionally, uncoated samples were measured and served as a reference. Each measurement was carried out at several angles of incidence (50°−80° in 1° steps) in two different ellipsometric zones (Nestler et al., 2012).

#### Chemical Composition

The elemental composition of the modified surfaces was analyzed by using an AXIS Supra X-ray photoelectron spectrometer from Kratos Analytical Ltd. (Manchester, United Kingdom). Measurements were performed using a monochromatic Al Ka X-ray source (1486.6 eV) operated at 150 W. Survey and core-level spectra were acquired using a pass energy of 80 eV. For each sample, an area of 250 μm was analyzed in duplicates on three different positions. During the analysis, the integrated charge neutralization system was activated for charge compensation. XPS measurements for PEI and PPI-G4 were performed with a PHI 5700 (Physical Electronics, United States). Here, survey and core-level spectra were acquired using pass energies of 117–187 eV. Charge neutralization was not necessary.

### Cell Biological Investigations

#### Cell Culture

Human osteoblast-like MG-63 cells (American Type Culture Collection ATCC^®^, CRL1427^TM^, Bethesda, MD, United States) were used; this cell line has been successfully applied as a model for studying cell-material interactions (Staehlke et al., 2019) and has similar characteristics to primary human osteoblasts (Clover and Gowen, 1994; Czekanska et al., 2014). The cells were cultured in Dulbecco’s Modified Eagle Medium (DMEM, 31966-021, Life Technologies Limited, Paisley, United Kingdom), with 10% fetal calf serum (FCS, Biochrom FCS Superior, Merck, Darmstadt, Germany) and 1% antibiotics (gentamicin, Ratiopharm, Ulm, Germany) at 37°C with 5% CO_2_/95% air atmosphere (Staehlke et al., 2018). All experiments were performed in passages 5–30, as the MG-63 cell physiology is known to remain stable over the entire range of these passages (Staehlke et al., 2019).

#### Cell Morphology

##### Scanning electron microscopy (SEM)

MG-63s (15,000 cells/cm^2^) were cultured for 24 h on the Ti arrays, washed three times with phosphate buffered saline (PBS, Sigma-Aldrich Chemie, Taufkirchen, Germany), fixed with 2.5% glutardialdehyde (Merck, Darmstadt, Germany) at 4°C over-night and rinsed with 0.1% sodium phosphate buffer (according to Sørensen, Merck, Darmstadt, Germany). The samples were then dehydrated through an ethanol series of 30, 50, 75, 90 and twice 100% (for 5, 5, 15, 10, and 10 min, respectively) and dried in a critical point dryer (K 850, EMITECH, Taunusstein, Germany). The samples were sputtered with gold for 50 s (15 nm, SCD 004, BAL-TEC, Wetzlar, Germany) and field emission scanning electron microscopy (FE-SEM, ZEISS Merlin VP compact, Carl Zeiss, Oberkochen, Germany) observations were taken with an acceleration voltage of 5 kV, a working distance of 5.6 mm and a high efficiency secondary electron detector (InlenseDuo for 2,000x, HE-SE2 detector for 100, 500x, and 5,000x).

##### Circularity

The circularity of the cells after 24 h could be evaluated with Photoshop CC 2017 using fluorescence microscopic images of fluoro-3-acetoxymethyl ester (fluo-3) stained cells (*n* ≥ 3 independent experiments á 15 cells). These images were also used for determining the cell area as basic values for the Ca^2+^ mobilization experiments (Supplementary Table S1). For the staining procedure see section “Intracellular Ca^2+^ mobilization.” A circularity value of 1.0 represents a perfect circle, whereas a value converging toward 0 indicates an elongated polygon.

#### Cell Viability

Cell viability was analyzed by an MTS assay, flow cytometry (both described in the Supplementary Material) and live/dead staining for selective surfaces representing negative, moderately and 2x highly positive surface charges (Ti, PPAAm, PPI-G4, and PDADMA, respectively). For live/dead staining a cell viability kit (L3224, Molecular Probes, Eugene, OR, United States) was used. This two-color fluorescence assay is based on the measurement of intracellular esterase activity of living cells (Calcein-acetoxymethyl ester, Calcein-AM) and the abrogation of the plasma membrane integrity of dead cells (Ethidium homodimer-1, EthD-1). While EthD-1 is excluded by the intact plasma membrane of living cells, it can enter cells with damaged cell membrane and binds to nucleic acids. After a cultivation period of 24 h MG-63s (80,000 cells/cm^2^) were washed carefully with PBS and incubated with a Calcein-AM/EthD-1 solution (1:1, 2 μM Calcein-AM, 4 μM EthD-1) for 20 min at 37°C in the dark. The cells were then rinsed with PBS and fixed with 4% paraformaldehyde (PFA, Sigma-Aldrich Chemie, Taufkirchen, Germany) at RT for 10 min. Samples were embedded on a coverslip with mounting medium Fluoroshield^TM^ (Sigma-Aldrich Chemie, Taufkirchen, Germany) containing 4′,6-diamidino-2-phenylindole (DAPI) and examined using the confocal laser scanning microscope LSM780 (Carl Zeiss, Jena, Germany) with a Plan-Apochromat 63x oil immersion objective (Carl Zeiss; zoom 1 and 2.5) and the ZEN software (ZEISS efficient navigation, ZEN 2011 SP4, black edition, Carl Zeiss).

#### Intracellular Ca^2+^ Mobilization

##### Pre-screening study

The Ca^2+^ mobilization process in MG-63s was specified with respect to the adenosine 5′-triphosphate (ATP) concentration for cell stimulation, Ca^2+^ origin and the presence of ATP receptors as provided in the Supplementary Material. The basal Ca^2+^ signal of MG-63s on Ti and PPAAm controls was validated by immunofluorescence. Therefore, 50,000 cells/cm^2^ were stained after 1 h with the Ca^2+^ indicator fluo-3 (5 μM, Life Technologies Corporation, Eugene, OR, United States) (Staehlke et al., 2015, 2018), Hoechst 33342 (1:1000, Life Technologies Corporation) and for images after 24 h additionally with 20 μl BacMam 2.0 reagent (CellLight^TM^ actin-RFP, Life Technologies Corporation) at 37°C in isotonic 4-(2-hydroxyethyl)-1-piperazineethanesulfonic acid buffer (HEPES) (Staehlke et al., 2015, 2018). Confocal laser scanning microscopy (LSM780, Carl Zeiss, Jena, Germany) was carried out with a Plan-Apochromat 63x oil immersion objective (Carl Zeiss, 1.40. Oil DIC M27) and the ZEN software (ZEISS efficient navigation, ZEN 2011 SP4, black edition, Carl Zeiss).

##### Ca^2+^ mobilization

Ca^2+^ mobilization experiments on different Ti surface modifications were performed according to Staehlke et al. (2015, 2018). In brief, 80,000 cells/cm^2^ were seeded onto the samples for 24 h and then loaded with 5 μM fluo-3 in Ca^2+^ containing HEPES buffer via a hypo-osmotic shock treatment. Ca^2+^ fluorescence signals of 10 single cells per surface over a time span of 480 s were recorded with a confocal laser scanning microscope LSM780 and the ZEN software (ZEN 2011 SP4, black edition) with the following settings: scan mode ‘time series‘ (1 frame every 2 s, 240 frames in total), maximum pinhole (15 airy unit, 13.5 μm section), gain 632, digital offset −3. First, the basal Ca^2+^ signal was recorded for 180 s. Then, the cells were stimulated with ATP (0.5 mM, SERVA Electrophoresis, Heidelberg, Germany), which indicates the reactivity of vital cells in dependence of the underlying chemical layer. At least three independent experiments per modification with 10 cells each for 240 analysis points per cell were investigated (=7,200 records per surface modification). The mean fluorescence intensity of the single cells (MFI_C_) was evaluated with the ZEN2 software (blue edition, version 2.0.0.0, Carl Zeiss). For this purpose, ten defined boxes were positioned on randomly chosen cells in the first image of the time series (one box per cell). Using the function ‘mean ROI’ (region of interest), the software analyzed the MFI of the boxes (MFI_ROI_) for 240 time points per cell. However, the MFI_ROI_ represents only a small sub-area of a cell (Supplementary Figure S4). Compared with a flat expanded cell, the Ca^2+^ signal of a spherical cell (more cell volume under limited area as cell height is larger) is concentrated on a smaller area, which leads to increased MFI_ROI_ values. Therefore, the MFI_ROI_ values were normalized to the mean area of cells after 24 h (A_C_) and the defined area of ROI (A_ROI_), assuming that MFI_ROI_ is independent of the ROI position in the cell. Accordingly, the A_C_ of 15 fluo-3 stained cells per surface were measured with the software Photoshop CC 2017 (*n* = 3 independent experiments, Supplementary Table S1). The MFI_C_ is described in the form:

$${\mathrm{MFI}}_{c}={\mathrm{MFI}}_{\mathrm{ROI}}\times \frac{A_{c}}{A_{\mathrm{ROI}}}$$

where MFI_C_ is the mean fluorescence intensity of cells at 0–480 s, MFI_ROI_ is the mean fluorescence intensity of the region of interest (ROI), A_C_ is the mean area of cells after 24 h (μm^2^), and A_ROI_ is the defined area of ROI (100 μm^2^). To calculate the mean *basal* fluorescence intensity of a cell (MFI_B_), MFI_ROI_ values of 0–170 s were used. In order to determine the MFI *after* ATP stimulation (MFI_A_), MFI_ROI_ values of 190–240 s were employed. The Ca^2 +^ mobilization (increase of the Ca^2 +^ signal = slope) was calculated by subtracting the MFI_B_ from MFI_A_.

### Statistics

Non-parametric Kruskal–Wallis followed by Dunn’s multiple comparisons test (or non-parametric Wilcoxon matched pairs signed-rank test) with the software GraphPad Prism version 6.05 for Windows (GraphPad Software, La Jolla, CA, United States) was conducted on the *p*-values < 0.05. Results are presented in (i) mean ± sem (standard error of the mean) for MFI_C_ values of the Ca^2+^ mobilization, (ii) in mean ± SD (standard deviation) for proliferation and cell area values, as well as for WCA, SFE, ζ-potential and XPS analyses, and (iii) in median with interquartile ranges (IQR) for cell circularity and MTS values.

## Results

### Physico-Chemical Characterization of Ti Surface Coatings

Results regarding layer thickness, wettability and surface charge (deduced from ζ-potentials) are given in Table 1. The chemical surface composition determined by XPS measurements is listed in Supplementary Table S2.

**TABLE 1:** Surface characteristics of chemically modified Ti (mean ± SD, *n* = 3).

Surface		Layer thickness [nm]	WCA [°]	SFE [mN/m] *dispersive polar*	ζ at pH 7.4 [mV]
Controls	Ti	9.0 + TiO_2_ (5.0)	87.4 ± 0.8	37.6 ± 1.4	**−87.5 ± 1.6**
				35.6 ± 1.1	
				2.1 ± 0.3	
	PPAAm	24.5	66.9 ± 1.7	46.21.5	**+7.1 ± 2.7**
				36.2 ± 0.6	
				10.0 ± 1.0	
Amino Polymers	APTES	5.0	88.1 ± 4.1	37.02.7	**+1.9 ± 1.5**
				35.1 ± 1.6	
				1.9 ± 1.0	
	PEI	18.0	27.6 ± 2.4	74.32.1	**+9.1 ± 2.6**
				48.8 ± 0.9	
				25.5 ± 1.1	
	PPI-G4	23.0	47.2 ± 4.3	62.43.6	**+50.2 ± 6.2**
				45.0 ± 1.2	
				17.4 ± 2.4	
ECM and Peptides	Col I	165.0	50.5 ± 4.3	58.63.5	**−2.8 ± 1.5**
				41.6 ± 1.1	
				17.0 ± 2.5	
	Matrigel	5.5	69.6 ± 6.9	46.75.4	**−43.3 ± 1.0**
				38.9 ± 2.1	
				7.8 ± 3.3	
	RGD	5.6	74.9 ± 1.2	43.01.8	**+1.7 ± 1.0**
				37.0 ± 1.2	
				6.0 ± 0.6	
Polyelectrolyte Multilayers	PSS	7.0	62.3 ± 2.7	49.32.3	**−88.8 ± 11.8**
				37.2 ± 0.8	
				12.1 ± 1.5	
	PDADMA	38.0	38.7 ± 3.2	62.03.3	**+51.4 ± 3.6**
				34.6 ± 1.3	
				27.3 ± 2.0	

#### Layer Thickness

The layer thickness of all modifications was less than or equal to 165 nm. The oxide layer of titanium surfaces was found to be 5 nm. The thinnest layer was achieved with RGD (0.6 nm) with an underlying APTES layer of 5.0 nm (in total 5.6 nm). The thickest layer was measured for Col I at 165 nm.

#### Wettability

The analysis demonstrated a change to more hydrophilic surfaces for all modifications except APTES, indicated by a decrease in WCA and an increase in SFE. Uncoated Ti surfaces as well as APTES-functionalized surfaces showed the highest WCA (with Ti 87.4° < APTES 88.1°), the lowest SFE under 40 mN/m and the lowest content of polar interactions with ≤2 mN/m. PEI-functionalized surfaces displayed the lowest WCA (27.6°) and consequently were the most hydrophilic surfaces showing the highest SFE values (74.3 mN/m) with polar interactions of 25.5 mN/m.

#### Zeta Potential

A less negative ζ-potential was achieved on almost all surfaces compared with uncoated Ti at −87.5 mV. Only the ζ-potential of PSS (−88.8 mV) was slightly lower. Further negative surface potentials were found for Matrigel (−43.3 mV). Col I substrates exhibit only a slightly negative ζ-potential (−2.8 mV). All other surfaces present a positive ζ-potential and can be classified as moderately and highly positive. Moderately positively charged surfaces include PPAAm, APTES, PEI and RGD (+7.1, +1.9, +9.1, and +1.7 mV, respectively), whereas highly positively charged surfaces comprise PPI-G4 and PDADMA (+50.2 and +51.4 mV, respectively). Here we sorted the investigated surfaces starting with the most negative ζ-potential: PSS < Ti < Matrigel < Col I < RGD < APTES < PPAAm < PEI < PPI-G4 < PDADMA.

### Cell Biological Investigations

The effect of positive and negative ζ-potential of chemically modified Ti surfaces on MG-63s behavior was observed and correlated.

#### Cell Morphology

**FIGURE 2:**
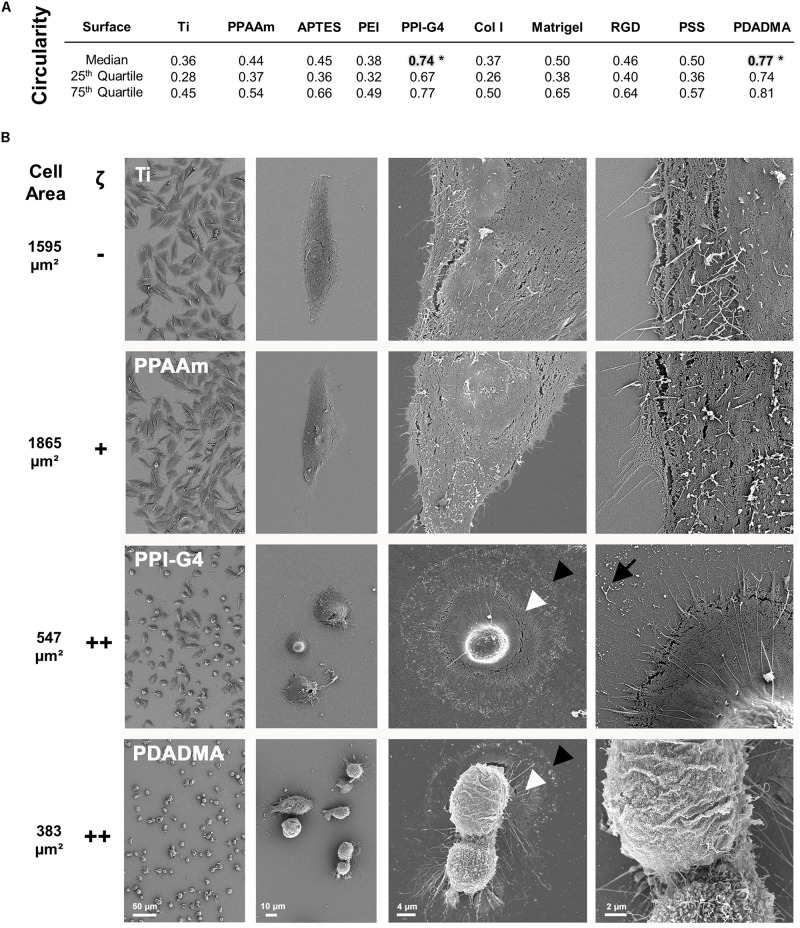
Morphology of MG-63s after 24 h on bare and functionalized Ti with negative (–), moderately positive (+) and highly positive (++) ζ-potential. **(A)** The cell circularity on highly positive surface charges reveals a morphologically significant difference to the other surfaces (*). A circularity value of 1.0 represents a perfect circle. (Statistics: Kruskal–Wallis and Dunn’s multiple comparison test, *p < 0.05; median ± IQR; *n* = 3 independent experiments). **(B)** Note the enormous decrease in cell area (from black arrowhead to white) on PPI-G4 and PDADMA. The PPI-G4 image (right) shows filopodia residues after the cell has retracted (arrow). On PDADMA different membrane structures with and without microvilli are visible (right image). (SEM images, magnification from left to right 100, 500, 2,000 and 5,000x, scale bars 50, 10, 4, and 2 μm, respectively, FE-SEM Merlin VP compact; mean cell area, *n* = 3 independent approaches, fluo-3 staining, LSM780).

The cell morphology on highly positively charged PPI-G4 and PDADMA surfaces was significantly changed to a more round shape (circularity 0.74 and 0.77, respectively) compared to all other surfaces with a polygonal shape (Figure 2A). High resolution SEM images revealed a decrease in cell area of MG-63s on PPI-G4 and PDADMA (Figure 2B, indicated by arrowheads), showing impaired cell morphology in contrast to well spread phenotypes on Ti and PPAAm. At 5,000x magnification, filopodia and membrane residues of cells on PPI-G4 are visible (arrow), whereas cells on PDADMA also show an altered membrane structure, as no microvilli are presented in individual cells.

#### Cell Viability

**FIGURE 3:**
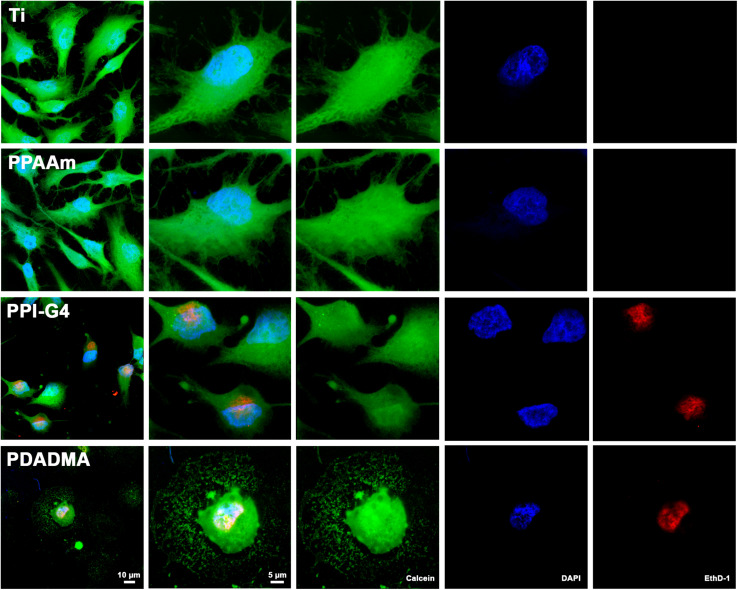
Cell viability of MG-63s after 24 h on Ti and PPAAm arrays representing negatively and moderately positive surface charges, as well as PPI-G4 and PDADMA, representing highly positive surface charges. Cells on PPI-G4 and PDADMA surfaces are stained with both calcein and EthD-1, indicating damaged plasma membranes. (Immunofluorescence images of live/dead staining; 63x objective with 1x and 2.5x zoom, scale bar 10 and 5 μm; nucleus in blue (DAPI), esterase activity of living cells in green (calcein), DNA of cells with abrogated membrane integrity in red (EthD-1); LSM780).

Fluorescence live/dead images displayed several cells on highly positively charged PPI-G4 and PDADMA surfaces that were positive for both Calcein and EthD-1 in contrast to Ti and PPAAm, suggesting a damaged plasma membrane (Figure 3). These images also confirmed the reduced cell area and circular shape on PPI-G4 and PDADMA after 24 h. In addition, we observed a decreased relative cell viability (Supplementary Figure S1) and cell proliferation (Supplementary Figure S2) on these surfaces compared with Ti and PPAAm, with a greater extent on PDADMA surfaces. While the amount of proliferative cells declined (with 64.1, 66.8, 51.8, and 24.6% for Ti, PPAAm, PPI-G4 and PDADMA, respectively), the cells increasingly remained in the G1 phase (34.0, 32.2, 47.5, and 73.7% for Ti, PPAAm, PPI-G4 and PDADMA, respectively).

#### Intracellular Ca^2+^ Mobilization

**FIGURE 4:**
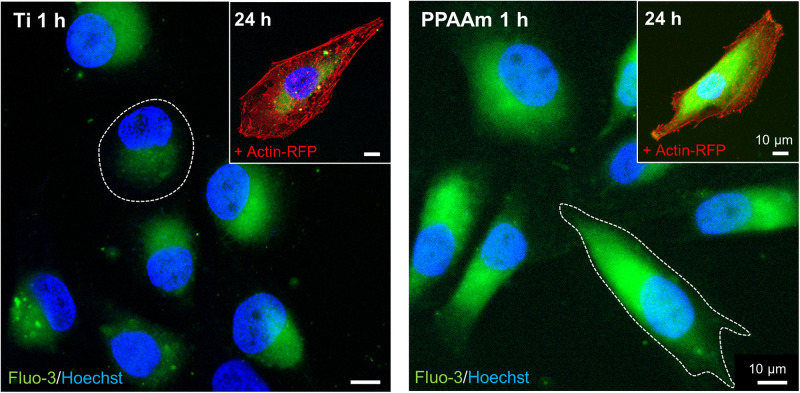
Basal Ca^2+^ signals in living MG-63s after 1 h and 24 h on the control surfaces Ti **(left)** and PPAAm **(right)**. The intracellular basal Ca^2+^ level on PPAAm is increased both after 1 and 24 h, suggesting higher cell function. While cells on PPAAm exhibit a larger cell area after 1 h (white dotted line), the area is balanced after 24 h. (Immunofluorescence images; 63x objective, scale bar 10 μm; nucleus in blue (Hoechst), Ca^2+^ ions in green (fluo-3), cytoskeleton in red (actin-RFP); LSM780).

The intracellular basal Ca^2+^ signals on the control surfaces Ti and PPAAm are depicted in Figure 4. The fluorescence images reveal an increased cell area on PPAAm after 1 h compared with the uncoated Ti control (white dotted lines), while cells are equally spread after 24 h (insert top right). The basal Ca^2+^ level was found elevated after 1 h as well as after 24 h on PPAAm compared with Ti. Consequently, these control surfaces were consistently included in the following Ca^2+^ mobilization experiments.

**FIGURE 5:**
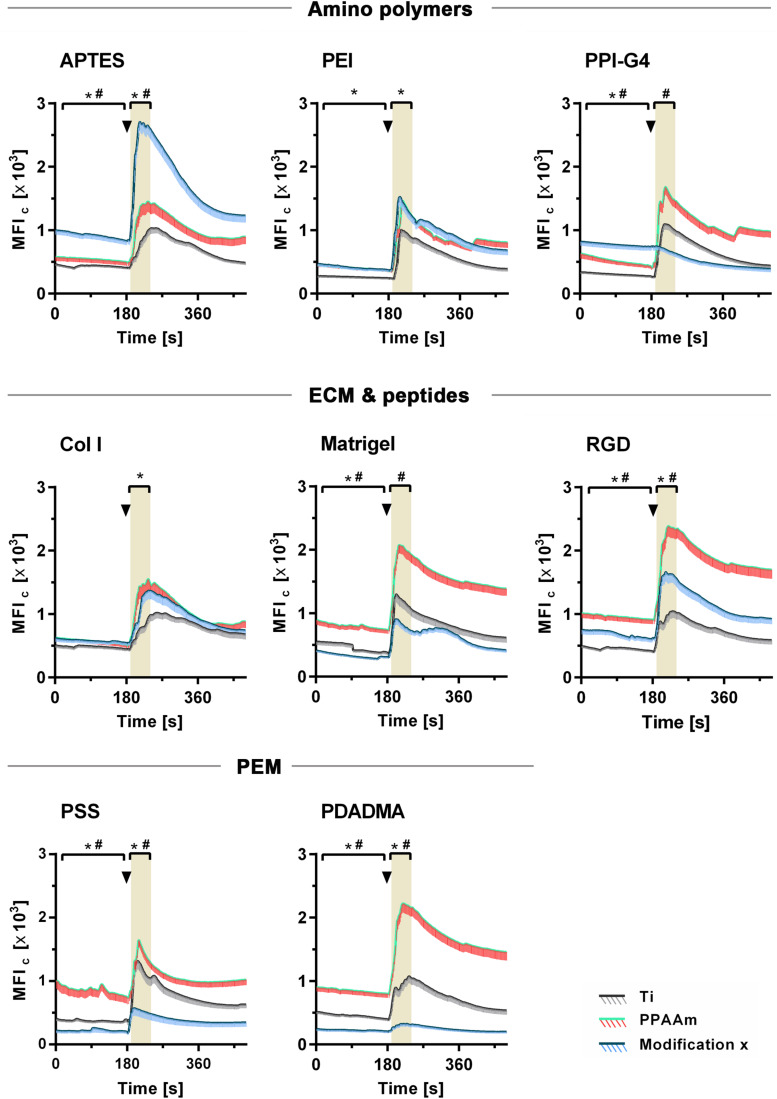
Dynamic Ca^2+^ mobilization in vital fluo-3 loaded MG-63s growing on amino polymers, extracellular matrix (ECM)/peptide motifs and polyelectrolyte multilayers (PEM) in the time frame 0–480 s. The positive control PPAAm (also an amino polymer) and the negative control Ti are embedded for the respective experiment. Shown are time courses of the mean fluorescence intensity of cells (MFI_C_) over 8 min: First, the basal Ca^2+^ signal is recorded for 180 s. At 180 s the cells are stimulated with ATP (arrowheads) and the Ca^2+^ signal is detected (LSM780, Carl Zeiss; ZEN-software). For statistical analyses of the basal MFI, values of the time points 0–170 s are used. For analyses of the MFI after ATP stimulation, values of the time points 190–240 s are used (highlighted in grey). MFI_C_ values are normalized to the mean cell area after 24 h. (Statistics: Kruskal–Wallis and Dunn’s multiple comparison test, *p* < 0.05; mean ± sem; *n* ≥ 3 independent approaches, per time point ≥ 30 cells; * indicates significance of modification x to Ti and ^#^ significance to PPAAm).

The influence of the individual Ti modifications on Ca^2+^ mobilization in vital cells is presented in Figure 5 (an overview of the time series with fluorescence images can be found in the Supplementary Figure S5). Shown are the whole time series of Ca^2+^ mobilization in MG-63 osteoblasts cultured 24 h on Ti modifications (blue curves) and the corresponding controls (gray curves = Ti, green/red curves = PPAAm). The exact values for MFI_B_, MFI_A_ and slope can be found in Table 2. Stimulation with ATP after 180 s resulted in a significantly lower Ca^2+^ mobilization in cells on PSS and PDADMA compared with their Ti controls, whereas the MFI_A_ of cells on Col I and PEI exceeded the Ti control significantly (Figure 5). Cells on Matrigel could not display a significantly different Ca^2+^ level upon stimulation than Ti. The MFI_A_ in cells on RGD-functionalized surfaces was significantly elevated compared with Ti, but below PPAAm. The highest Ca^2+^ mobilization after ATP stimulation was found in cells on APTES. Cells cultured on PDADMA surfaces exhibited the lowest mobilization rate, while cells on PPI-G4 surfaces could not mobilize Ca^2+^ ions at all.

**TABLE 2:** Ca^2+^ mobilization results related to surface characteristics of chemically modified Ti (mean ± SD, *n* = 3 for surface characteristics; mean ± sem, *n* ≤ 3 for Ca^2+^ values).

Surface	PSS	Ti	Matrigel	Col I	RGD	APTES	PPAAm	PEI	PPI-G4	PDADMA
ζ [mV]	−88.812	−87.52	−43.31	−2.81	1.71	1.91	7.13	9.13	50.26	51.44
MFI_B_	216.440	445.118	329.620	575.344	683.660	918.572	656.632	41235	769.863	221.528
MFI_A_	523.4107	104053	79172	1037.597	1484.3123	2310.9157	1461.458	1232.4104	691.854	300.637
Slope	307.1134	594.971	461.3104	462.2105	800.7126	1392.4169	804.853	820.4138	−78.017	79.118
WCA [°]	62.33	87.41	69.67	50.54	74.91	88.14	66.92	27.62	47.24	38.73
SFE [mN/m]	49.32	37.61	46.75	58.63	43.02	37.03	46.21	74.32	62.44	62.03

**FIGURE 6:**
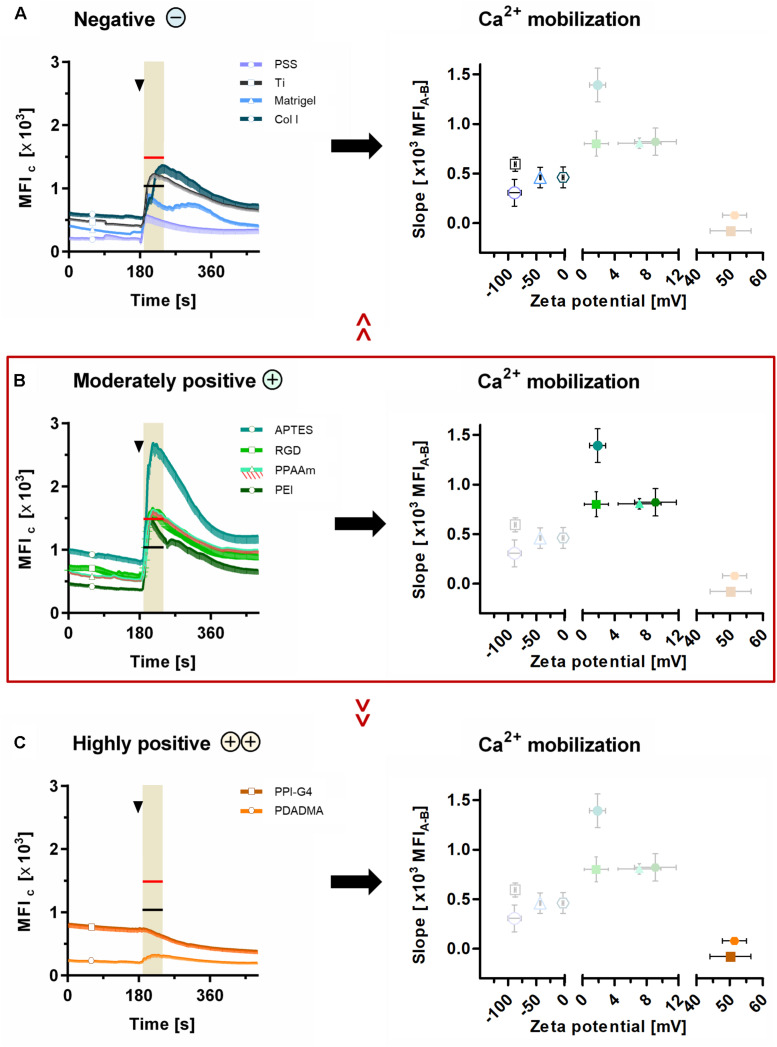
Ca^2+^ mobilization results related to ζ-potential. Presented are (i) the mean fluorescence intensity of cells (MFI_C_) after 24 h on surfaces with **(A)** negative, **(B)** moderately positive and **(C)** highly positive ζ-potentials compared with the mean fluorescence intensity after ATP stimulation (MFI_A_) of cells on Ti (black lines at time points 170–240 s in highlighted area) and PPAAm (red lines at time points 170–240 s in highlighted area), and (ii) the correlation of the Ca^2+^ mobilization (slope) with the materials’ ζ-potentials. Note that the slope of the Ca^2+^ mobilization curve is strongly dependent on ζ-potential values. Here, the surfaces with moderately positive ζ-potentials indicate a beneficial impact. (Ca^2+^ signaling: mean ± sem, *n* ≥ 3 independent approaches, per time point ≥ 30 cells for modifications and 190 cells for Ti and PPAAm controls, arrowheads at 180 s indicate ATP stimulation). PSS, poly(styrene sulfonate); Ti, titanium; Col I, collagen-type I; APTES, (3-aminopropyl)triethoxysilan; RGD, peptide sequence Arg-Gly-Asp; PPAAm, plasma polymerized allylamine; PEI, poly(ethylene imine); PPI-G4, poly(propylene imine) dendrimer generation 4; PDADMA, poly(diallyldimethylammonium chloride).

These results could be correlated with the ζ-potential of the substrates, as shown in Figure 6. Here, the sections 5A-C are classified into negative, moderately and highly positive according to ζ-potential values, starting with the most negative and ending with the most positive value. With this alignment the diminished Ca^2+^ mobilization in cells on surfaces with negative (Figure 6A) and highly positive ζ-potential (Figure 6C) compared with surfaces with moderately positive ζ-potential (Figure 6B) could be clearly demonstrated. Cells on surfaces with negative ζ-potentials of −88.8 to −43.3 mV displayed significantly lower Ca^2+^ levels after ATP stimulation than PPAAm, the positively charged control (red line in highlighted area = MFI_A_ of PPAAm) (Figure 6A). Exclusively, Col I-functionalized surfaces with an almost neutral ζ-potential of −2.8 mV lacked significance to PPAAm. The absolute slope from basal Ca^2+^ level to the level after ATP stimulation reached a maximum of 594.9 MFI_C_ on negatively charged surfaces (Table 2).

Materials revealing a moderately positive ζ-potential of ∼1 to 10 mV consistently exhibited significantly higher Ca^2+^ levels after stimulation than Ti, the negatively charged control (black line in highlighted area = MFI_A_ of Ti, Figure 6B). Here, the Ca^2+^ mobilization ranged between 800.7 and 1392.4 MFI_C_ (Table 2).

On substrates presenting highly positive ζ-potentials (∼ + 50 mV), MG-63s indicated an impaired Ca^2+^ mobilization with MFI_A_ values significantly lower than PPAAm and Ti (Figure 6C). The maximum Ca^2+^ level increase was 79.1 MFI_C_ (Table 2).

Furthermore, a correlation of Ca^2+^ mobilization data with the measured surface properties for wettability (WCA and SFE) could not be identified (Table 2). There were both less and more hydrophilic surfaces in the range of cells showing a stronger ability to mobilize intracellular Ca^2+^ (e.g., ∼90° for APTES and ∼30° for PEI) as well as a lower ability for Ca^2+^ mobilization (e.g., ∼90° for Ti and ∼40° for PDADMA). However, osteoblasts behave differently with respect to their Ca^2+^ dynamics. A similar statement can be formulated regarding the SFE values. Here, the highest SFE values (Col I < PDADMA < PPI-G4 < PEI with 58.6, 62.0, 62.4, and 74.3 mN/m, respectively) did not automatically lead to the best Ca^2+^ mobilization results, especially noticeable for PPI-G4 and PDADMA, which displayed the lowest slope.

## Discussion

Cells in living bone tissue are surrounded by an electrically charged, organic/inorganic solid that is permeated by a flow of ionic solution through an intricate channel system (Chakkalakal, 1989). Consequently, it is not surprising that bone cell physiology is also affected by electrical cues when cultivating cells on artificial material surfaces. Cell-material interactions are highly complex and require more systematic investigations regarding surface charge. It is certain that electrostatic forces are crucial for cellular attachment via focal adhesion to material surfaces (Metwally and Stachewicz, 2019) influencing the further fate of cells. Earlier studies have mostly dealt with a limited number of surface charges [e.g., only negative surfaces (Altankov et al., 2003), one positive compared with several negative surfaces (Chang et al., 2014), or only one negative, neutral and positive surface charge (Iwai et al., 2013)] disallowing detailed statements. It has been reported that materials with positive surface potentials have a beneficial effect on cell viability as well as on adhesion and spreading. But most studies lacked a clear determination of the surface potential (Lee et al., 1994; Bacakova et al., 1996; Webb et al., 1998; Lesný et al., 2006). Hence, the underlying mechanism controlling cell activities using the materials’ surface charge is still not fully understood in bone tissue engineering.

In the present study, the behavior of osteoblastic MG-63s via intracellular Ca^2+^ mobilization was systematically investigated on different Ti modifications and correlated with the materials’ ζ-potential as a parameter for the electric surface property. We used unmodified Ti (−87.5 mV) and PPAAm coated Ti (+7.1 mV) as controls with negative and positive ζ-potential, respectively, as published before (Mörke et al., 2017; Nebe et al., 2019), knowing that the PPAAm surface modification enhances cell physiology concerning adhesion, spreading, motility (Rebl et al., 2010), actin filament network (Rebl et al., 2016), viability and Ca^2+^ mobilization (Moerke et al., 2018; Staehlke et al., 2018), as well as the implant osseointegration (Hoene et al., 2010). Cells bind to this positively charged plasma polymer layer via electrostatic forces, as it is known that osteoblasts (Finke et al., 2007), epithelial cells and chondrocytes (Cohen et al., 2004) express i.a. the negatively charged hyaluronan as a spherical shell around the cells (Zimmerman et al., 2002). However, the question arose whether positive ζ-potentials generally lead to a beneficial cellular outcome.

### Physico-Chemical Characterization of Ti Surface Coatings

Therefore, we successfully prepared nine chemical modifications of Ti substrates with (i) amino polymers, (ii) ECM/peptide motifs, and (iii) PEM, resulting in a broad range of ζ-potentials. The materials’ characteristic ζ-potential, WCA, SFE, layer thickness (Table 1) and chemical composition (Supplementary Table S2) were evaluated.

The layer thickness was in the nanometer range (max. 165 nm). The APTES-modified surface layer was similar in thickness to the PEI and PPI-G4 layers. These layers are SAM arrangements, whereby the smaller thickness of the APTES layer correlates with the shorter molecular length of the APTES molecule compared with both the PEI and PPI-G4 molecule lengths. The molecular length of the RGD-conjugated APTES molecule is slightly longer than the APTES molecule itself. However, it is unlikely that an RGD molecule is coupled to every amino group on APTES. It therefore seems possible that individual RGD molecules are not necessarily aligned completely orthogonal to the surface as it is the case in a perfect SAM arrangement. In addition, interactions like hydrogen bonding between amino groups on APTES and residual amino acids of the RGD may possibly lead to a “bending” of the RGD molecules and consequently to a reduction of the overall layer thickness. Regarding PPAAm coatings, it must be emphasized that PPAAm is a very complex, highly cross-linked structure, which was presented here only in a very simplified and model-like schematic manner (see Figure 1). The thickness of PPAAm, as well as Col I, Matrigel, and the polyelectrolyte multilayer coatings is mainly determined by the individual material properties and the process parameters.

The wettability analysis demonstrated a change to more hydrophilic surfaces. There is evidence that mammalian cells favor modestly hydrophilic surfaces displaying water contact angles between 40° and 65° (Rebl et al., 2012; Ahn et al., 2014; Gentleman and Gentleman, 2014). However, Gentleman and Gentleman (2014) reviewed that studies often lack a clear trend of cell behavior with wettability. They rather assumed SFE to be the more relevant feature for cellular behavior. This could not, however, be confirmed here. We could neither find a connection between WCA and SFE to the surface charge which is consistent with Olsson et al. (1995), nor a correlation with the cell behavior.

The ζ-potential could rather be the pivotal parameter for controlling cell physiology in osteoblasts, as our results indicate. In whole bone tissue negative ζ-potentials could be determined (Skalak and Shu, 1986; Otter et al., 1988; Chakkalakal, 1989). In this study, ζ-potentials of the investigated surface modifications were identified in the range of −90 to −3 mV (PSS < Ti < Matrigel < Col I), +1 to +10 mV (RGD < APTES < PPAAm < PEI) and ∼+50 mV (PPI-G4 < PDADMA). For the first time, we were able to classify the ζ-potential into three categories with regard to an improved cell response on the respective surfaces and introduce the following terms: negative < moderately positive > highly positive.

### ζ-Potential – A Parameter for Surface Charge – Can Strongly Influence Cellular Behavior

It is known that physiology features of MG-63s such as actin cytoskeleton organization (Staehlke et al., 2015), viability (Staehlke et al., 2018) or proliferation (Labelle et al., 2007) are reflected by intracellular Ca^2+^ dynamics. However, to our knowledge there is no literature on intracellular Ca^2+^ dynamics in the context of the ζ-potential of biomaterials other than our previously published study comparing surfaces with negative ζ-potential with positive charges of plasma polymer nanolayers (PPAAm). The question remained, if positive charges in general promote the cellular outcome.

In order to gain deeper insights into the effect of surface charges on Ca^2+^ dynamics, we analyzed the intracellular Ca^2+^ signals before (MFI_B_) and after ATP stimulation (MFI_A_) on surface modifications with various charges using confocal laser scanning microscopy (Figure 5 and Supplementary Figure S5). Additionally, cell morphology (Figure 2), cell viability (Figure 3 and Supplementary Figure S1) and cell proliferation (Supplementary Figure S2) were investigated on selected surfaces. In the following, our results are discussed regarding negative, moderately and highly positive ζ-potentials.

### Negative ζ-Potential (−90 to −3 mV)

On our surfaces with negative ζ-potential we found evidence of reduced Ca^2+^ mobilization after 24 h compared with surfaces with moderately positive ζ-potential. On PSS terminated PEM surfaces, MG-63s showed one of the lowest Ca^2+^ slopes after 24 h. Other studies documented that hMSC cell numbers after 2, 5, and 7 days were consistently lower on PSS terminated PEM films than on control surfaces (Liao et al., 2010). However, PSS ending films exhibited good biocompatibility, as shown for SaOS-2 cells (cytokine IL-8 production) (Tryoen-Tóth et al., 2002), at least for films consisting of not more than six polyelectrolyte layers (Arias et al., 2016).

### Moderately Positive ζ-Potential (+1 to +10 mV)

Surfaces in the moderately positively charged range were found to promote the cell physiology via Ca^2+^ mobilization. Cells can access their intracellular Ca^2+^ stores more effectively after an external stimulus (here: ATP) than cells on negatively charged substrates, suggesting higher cell activities.

Ravenscroft-Chang et al. (2010) loaded primary cardiac myocytes growing on (3-trimethoxysilylpropyl) diethylenetriamine (DETA) silane modified and fluorinated glass substrates with fura-2 AM and induced a Ca^2+^ signal by electrical stimulation (1 HZ, 6 V, 5 ms/pulse). Cells exhibited a significantly higher Ca^2+^ level, as shown in higher amplitude and duration. However, the authors did not determine the ζ-potential of their surfaces. But a slightly positive ζ-potential at pH 7.4 of such DETA amino silane treated glass surfaces was measured earlier by Metwalli et al. (2006) supporting the idea of higher cellular activity on the basis of moderately positive charges.

For PEI functionalized surfaces it is known that cytotoxicity can exist depending on the molecular weight and concentration (Brunot et al., 2007). However, in studies with surfaces derived from low molecular weight PEI, as used here (25 kDa), no cytotoxic effects were reported for fibroblasts up to 7 days (Hernandez-Montelongo et al., 2017). PEI modification could further improve proliferation and function of MG-63s (Liu et al., 2009), as indicated here by an increased Ca^2+^ slope.

RGD peptide motifs covalently bonded to Ti surfaces promote osteoblast attachment and survival (Secchi et al., 2007). El-Ghannam et al. (2004) showed that APTES-RGD coatings provided the optimal surface for cell adhesion, spreading, and cytoskeletal organization for MC3T3-El cells, better than RGD and APTES alone. It must be taken into consideration that surfaces modified with RGD (as well as Col I and Col IV and laminin containing Matrigel) can trigger Ca^2+^ mobilization through integrin mediated “outside-in” signal transduction during cell adhesion (Sjaastad and Nelson, 1997; Boraschi-Diaz et al., 2017). After 24 h cultivation, however, integrin signaling may no longer be that influential in this case, as no additional stimulus such as mechanical stress (Pommerenke et al., 2002) was applied. Otherwise an increased Ca^2+^ mobilization on these ECM coated surfaces would be expected. Here, we found that APTES alone was capable of mobilizing Ca^2+^ in MG-63s even more than PPAAm – surfaces that do not present integrin ligands.

Lee et al. (2018) presented ATPES-treated PDMS concave microwell surfaces to cortical neurons which revealed optimal surface conditions additionally supporting a spheroid formation. The authors also stimulated the cells with KCl for spontaneous calcium transients (fluo-4 AM), observing typical temporal Ca^2+^ responses in all chemically modified microwells based on APTES (APTES, APTES-Laminin, APTES-poly-L-lysine, APTES-carbon nanotubes).

### Highly Positive ζ-Potentials (∼+50 mV)

On PPI-G4 and PDADMA surfaces with highly positive ζ-potentials we discovered a restricted cell viability, reflected in an impaired Ca^2+^ mobilization and a disturbed cell morphology and proliferation after 24 h, indicating that cells cannot tolerate such ζ-potentials.

Koch et al. (2018) produced the 11-amino acid self-assembling peptides P11-8 and P11-28/29 hydrogels exhibiting ζ-potentials of ∼ + 20 mV and ∼ + 60 mV at pH 7.0, respectively. HPDLF and HCO cells on highly positive P11-28/29 surfaces showed reduced cell area after 24 h, while cells on P11-8 displayed also enhanced osteogenic differentiation (ALP activity) compared with hydrogels containing negatively charged glutamic acids. Similarly, Kidambi et al. (2004) reported that primary hepatocytes were unable to remain attached to PEM films with PDADMA as the outermost layer. Inactive cells completely lifted off the surface by day 7 where liver-specific functions approached zero. In our previous study (Gruening et al., 2020) we found an impeded cell spreading dynamic on PDADMA surfaces after an initial tendency to spread better than Ti. The longer the influence of strongly positive charges on the cells, the more the cells were hampered.

Guo et al. (2018) pointed out that on positively charged non-cross-linked PEI films (ζ-potential: +50 and +62 mV) more fibroblasts could adhere within the first 24 h compared with negatively charged polymer films. But, on the second day, cells on the negatively charged surfaces proliferated well, in contrast to those on samples with high ζ-potentials. The authors argued that this was due to the reported cytotoxicity of PEI. But by cross-linking these PEI films, the high ζ-potential was reduced to ∼ + 20 mV and cells proliferated on all surfaces.

During the cell cycle, checkpoints in G1 and G2 (gap) phases prevent cells from entering into the next phase in case of cell injury e.g., due to accumulation of reactive oxygen species (ROS) (Muller et al., 2007) or inadequate environmental conditions such as bacterial infection (Kato et al., 2008), hypoxia (Zou et al., 2015) or withdrawal of nutrition (Blagosklonny, 2011). Cells which do not pass the safety controls proceed with the non-proliferative G0/G1 phase in quiescence (cell cycle arrest) or even permanent senescence (Blagosklonny, 2011; Terzi et al., 2016). We found an increasing cell amount in the G1 phase on highly positive surfaces, leading to a decline of cellular proliferation. In further experiments it is to be examined whether the high surface charges lead to an elevated ROS production as stress response and thus to a cell cycle arrest, allowing cells to cope with their environment.

However, the high surface charge leads to an intense interaction with the negatively charged cell surface (Rebl et al., 2016), which is further considered to cause damaged lipid bilayers due to new vesicular structures around the cationic molecules which create holes in the cell membrane (Mecke et al., 2005). This could be supported by the live/dead staining, demonstrating an abolished membrane integrity in still vital cells on PPI-G4 and PDADMA.

The adverse effects of highly positive surface charges deduced from the ζ-potential might also be indirectly linked to protein adsorption, since it has been published that the protein adsorption is strongly affected by the ζ-potential (Krajewski et al., 1998; Lehnfeld et al., 2020) influencing the protein population (Shelton et al., 1988) and orientation (Norde and Lyklema, 1991; Antonini et al., 2014). Therefore, a further correlation between protein adsorption and ζ-potential of different surface modifications with well-defined effects on the cell behavior is of great interest.

## Conclusion

This is the largest systematic study so far documenting the importance of the materials’ surface charge deduced from ζ-potential for the intracellular Ca^2+^ dynamics and viability of human osteoblasts. Positive charges offer unique cues that induce an intensive cell response. But interestingly, we identified that cells favor only a certain range of moderately positive surface charges and not a positively charged surface in general. The ζ-potential appears to be a key property of biomaterial surfaces dominating the relevance of wettability (WCA, SFE), and should be considered in biomaterial coating design in tissue engineering and dental and orthopedic implantology.

## Data Availability Statement

The datasets presented in this study can be found in online repositories. The names of the repository/repositories and accession number(s) can be found below: Mendeley data 10.17632/h8rs6v4dgt.1.

## Author Contributions

MG: conceptualization, investigation, validation, visualization, and original draft preparation. SN: PEM preparation. PN: layer thickness measurement and review and editing. KF: XPS measurement and PPAAm preparation. MD: APTES and RGD preparation. JL: PEI and PPI-G4 preparation and review and editing. MS: visualization and review and editing. CH and RM: review and editing and funding acquisition. SS: ATP receptor immunofluorescence and review and editing. JBN: conceptualization, review and editing, project administration, and funding acquisition. All authors contributed to the article and approved the submitted version.

## Conflict of Interest

The authors declare that the research was conducted in the absence of any commercial or financial relationships that could be construed as a potential conflict of interest.

## Abbreviations

AM: acetoxymethyl ester
APTES: (3-aminopropyl)triethoxysilan
ATP: adenosine 5‘-triphosphate
Col I: collagen-type I
Col IV: collagen-type IV
DAPI: 4′,6-diamidino-2-phenylindole
DETA: (3-trimethoxysilylpropyl)diethylenetriamine
DMEM: Dulbecco’s Modified Eagle Medium
DMSO: dimethyl sulfoxide
ECM: extracellular matrix
EDC: *N*-(3-dimethylaminopropyl)-*N’*-ethylcarbodiimide hydrochloride
Ent: entactin
EthD-1: Ethidium Homodimer 1
HSPG: heparan sulfate proteoglycans
Lam: laminin
MES: 2-(*N*-morpholino) ethanesulfonic acid
MFI_C_: mean fluorescence intensity of cells
MFI_B_: basal mean fluorescence intensity
MFI_A_: mean fluorescence intensity after ATP stimulation
MTS: 3-(4,5-dimethylthiazol-2-yl)-5-(3-carboxymethoxyphenyl)-2-(4-sulfophenyl)-2H-tetrazolium salt
NHS: *N*-hydroxysuccinimide
OeTS: 7-octenyltrichlorosilane solution
PAH: poly(allylamine hydrochloride)
PAMAM: poly(amidoamine)
PBS: phosphate buffered saline
PDADMA: poly(diallyldimethyl ammonium chloride)
PEI: poly(ethylene imine)
PEM: polyelectrolyte multilayer
PES: polyethersulfone
PFA: paraformaldehyde
PP: proliferative phase
PPAAm: plasma polymerized allylamine
PPI-G4: poly(propylene imine) dendrimer generation 4
PSS: poly(styrene sulfonate)
RGD: peptide sequence Arg-Gly-Asp
ROI: region of interest
ROS: reactive oxygen species
RT: room temperature
SAM: self-assembled monolayer
SEM: scanning electron microscope
SFE: surface free energy
SPDP: *N*-succinimidyl -3-(2-pyridyldithio) propionate
TESPSA: 3-(triethoxysilyl)propyl succinic acid anhydride
Ti: titanium
WCA: water contact angle
XPS: X-ray photoelectron spectroscopy
ζ: zeta potential.
