# Supplementary material for: Enhancement of Intracellular Calcium Ion Mobilization by Moderately but Not Highly Positive Material Surface Charges
Source: Front Bioeng Biotechnol. 2020 Sep 8;8:1016. doi: 10.3389/fbioe.2020.01016 (PMC7505933; doi:10.3389/fbioe.2020.01016)
Supplement: Supplementary file 1 [file Data_Sheet_1.pdf]

Supplementary Material

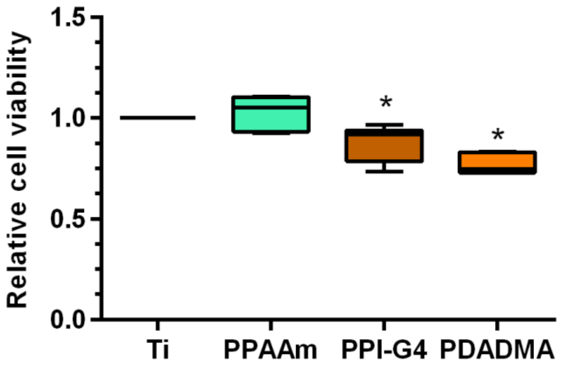

Supplementary Figure S1. Relative cell viability of MG-63s on highly positively charged PPI-G4 and PDADMA surfaces compared with Ti (negatively charged) and PPAAm (moderately positively charged) after 24 h. Presented are cell metabolism values based on MTS measurements. Values are related to the cell density deduced from crystal violet staining and normalized to the Ti control. Note that the relative cell viability of MG-63s on highly positively charged surfaces is significantly decreased in contrast to Ti and PPAAm. (Statistics: Nonparametric Wilcoxon matched pairs signed-rank test, *p > 0.05; median ± IQR with maximum and minimum; n = 3 independent experiments, Anthos reader).

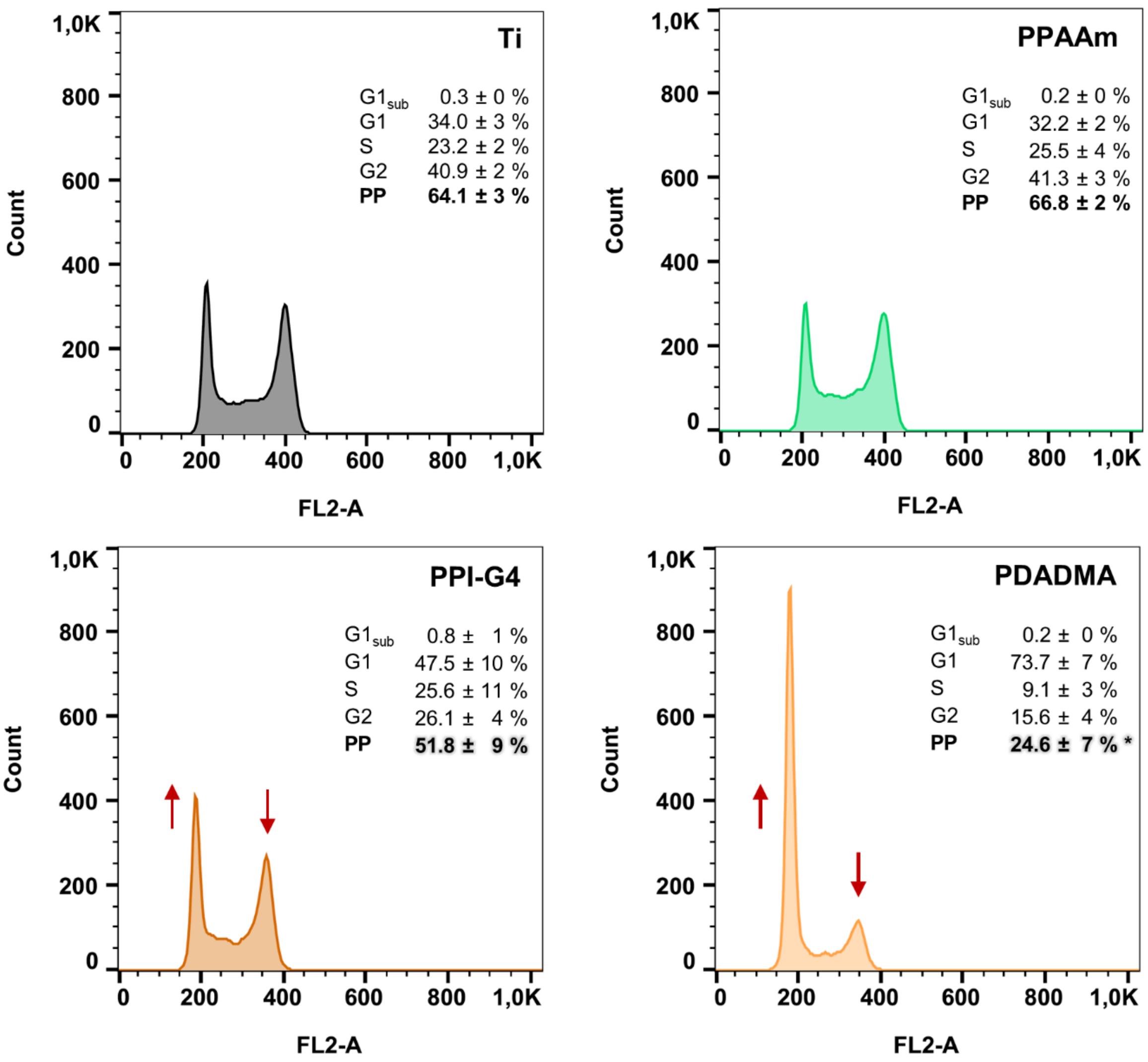

Supplementary Figure S2. Cell proliferation of MG-63 osteoblasts on highly positively charged PPI-G4 and PDADMA surfaces compared with the controls Ti (negatively charged) and PPAAm (moderately positively charged) after 24 h. Demonstrated are representative examples of cell cycle histograms of the respective surfaces and the proliferative phase (PP = S + G2), G1 and apoptosis peak ($G1_{sub}$) calculated from three independent experiments. Note the reduction of proliferative cells due to highly positive surface charges, while the cells increasingly remain in the G1 phase (indicated by red arrows). Late apoptotic events ($G1_{sub}$) could not be detected for all surfaces after 24 h. (Statistics: Nonparametric Kruskal-Wallis + Dunn´s multiple comparisons test, $p > 0.05$, * significant decrease compared with Ti and PPAAm; mean ± SD; n = 3, flow cytometer FACSCalibur).
(Abbreviations: PPI-G4, poly(propylene imine) dendrimer generation 4; PDADMA, poly(diallyldimethylammonium chloride, PPAAm, plasma polymerized allylamine; PP, proliferative phase, G, gap; S, synthesis phase).

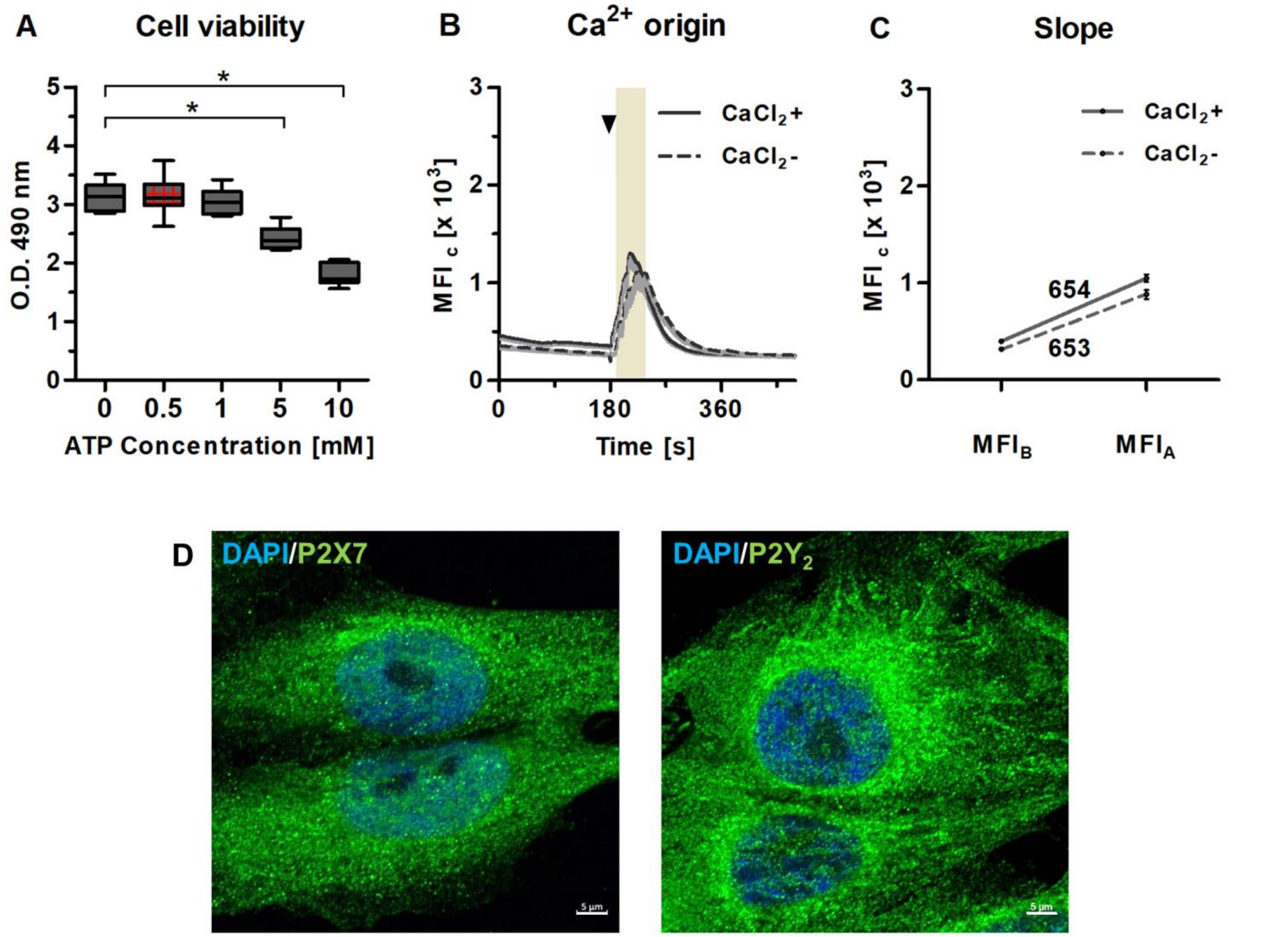

Supplementary Figure S3. Intracellular Ca^{2+} mobilization via ATP receptors - a pre-screening study. (**A**) ATP concentration: Cell viability (MTS) of MG-63s after 20 min incubation with different ATP concentrations. A non-toxic concentration of 0.5 mM was used for all Ca^{2+} mobilization experiments (red grid lines). (Statistics: Mann-Whitney U-test, $*p < 0.05$; median ± IQR and minimum and maximum; n = 3). (**B**) Ca^{2+} source: MFI_C (Mean fluorescence intensities of cells) of intracellular Ca^{2+} in cells on Ti surfaces in $CaCl_2$ containing HEPES ($CaCl_2$ +) and HEPES without $CaCl_2$ ($CaCl_2$ -).There is no significant difference between $CaCl_2$ + and $CaCl_2$ - for MFI_B (basal MFI = 0–170 s) and MFI_A (MFI after stimulation with ATP = 190–240 s, highlighted area). The arrow indicates the addition of ATP after 180 s. (Statistics: Mann-Whitney test, $*p > 0.05$; mean ± sem; n = 3, LSM780). (**C**) Ca^{2+} mobilization: The slope from MFI_B to MFI_A is the same for $CaCl_2$ + (654 MFI_C) and $CaCl_2$ – (653 MFI_C). (**D**) ATP receptors: Fluorescence images of ATP receptor types $P2X_7$ (left) and $P2Y_2$ (right) (green) in MG-63s (cell nucleus, blue). (Magnification 63x, scale bars 5 µm, LSM780).

Role of ATP receptors: $P2Y_2$ is an ATP-sensitive G-protein coupled receptor. External ATP stimulation of MG-63s activates the phospholipase-C pathway, triggering the osteoblasts to mobilize their intracellular Ca^{2+} storage from the endoplasmic reticulum, leading to increasing cytoplasmic Ca^{2+} levels. An extracellular Ca^{2+} influx via ATP-sensitive ligand-gated ion channels (e.g. $P2X_7$) appears to be of secondary importance in this case.

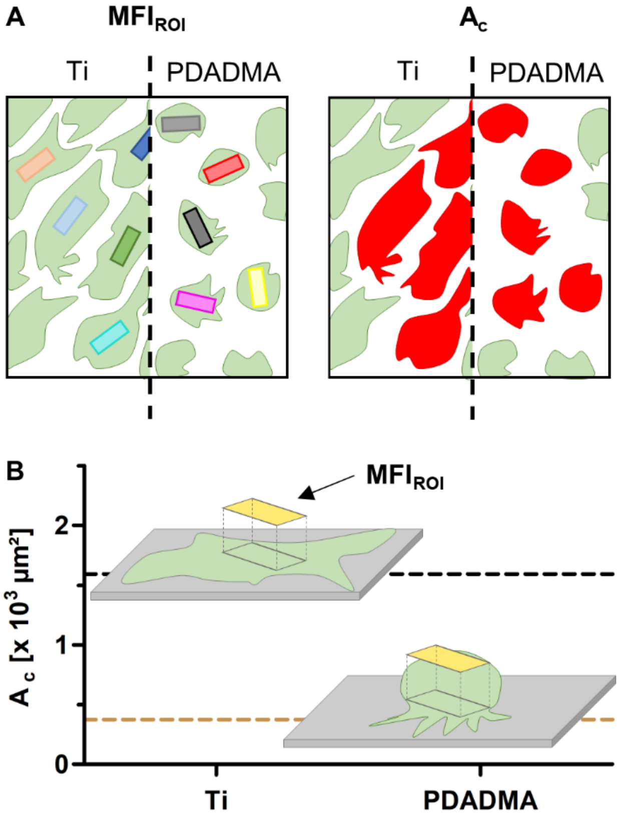

Supplementary Figure S4. Calculation example of the mean fluorescence intensity of cells (MFI_C) on Ti and PDADMA. (**A**) The MFI of the 'region of interest' (MFI_{ROI}) is determined by boxes (colored rectangles) with defined areas of 100 µm² (A_{ROI}) placed on randomly selected fluo-3 stained cells (green). The cell area A_C (red) is measured from flat cells on Ti and spherical cells on PDADMA. (**B**) The Ca^{2+}-signal of a spherical cell on PDADMA is concentrated on a smaller area, which results in misleadingly high MFI_{ROI} values.

Supplementary Table S1. Cell area of MG-63s after 24 h on chemically modified Ti: The basic values for MFI_C calculation were determinated using microscopic images of fluo-3 stained cells (mean ± SD, n = 3).

Surface	Ti	PPAAm	APTES	PEI	PPI-G4	Col I	Matrigel	RGD	PSS	PDADMA
**Cell area [µm²]**	1595 ± 108	1865 ± 175	1623 ± 131	1621 ± 169	547 ± 45	1836 ± 210	1553 ± 241	1568 ± 207	1181 ± 90	383 ± 42

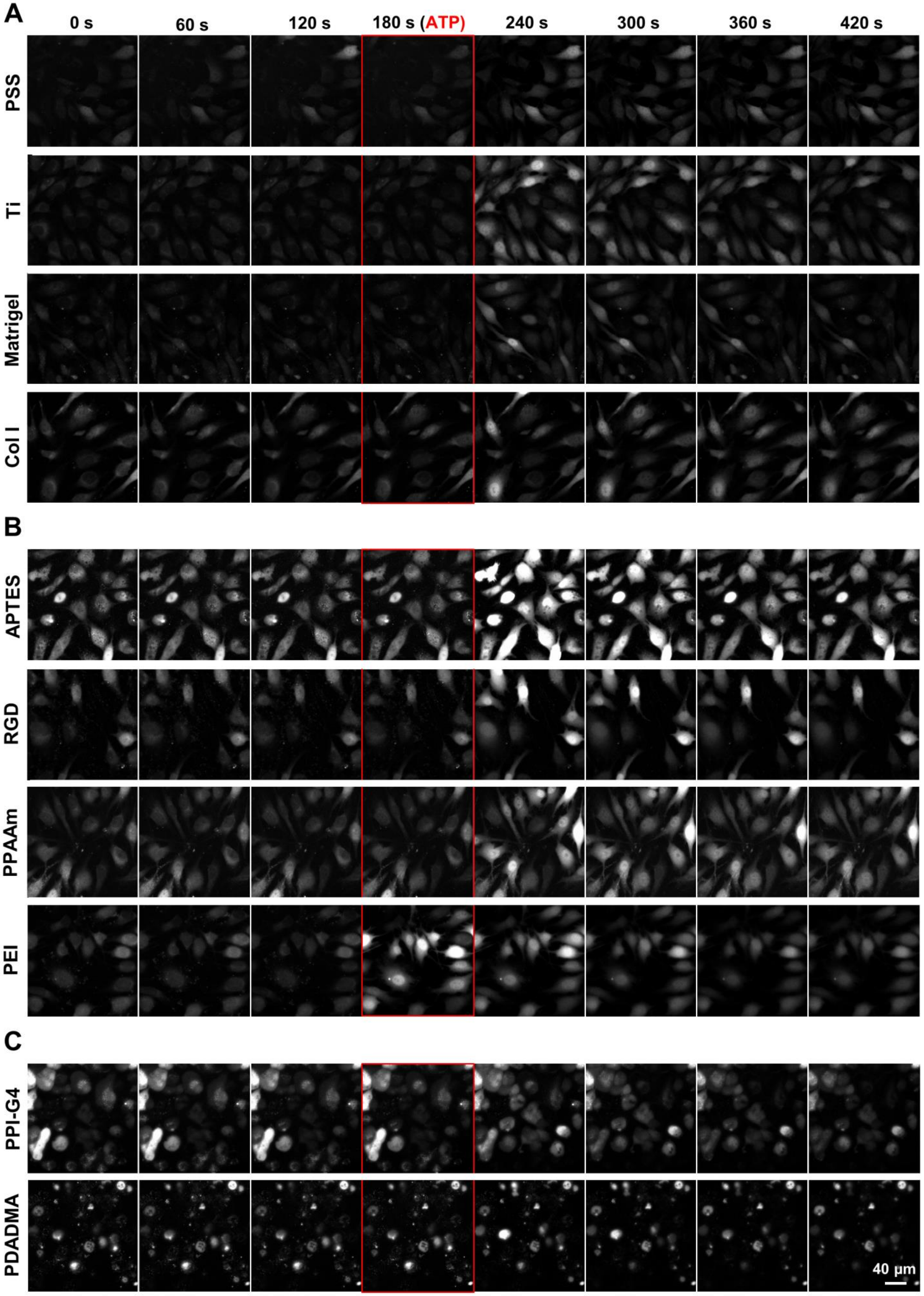
A
0 s
60 s
120 s
180 s (ATP)
240 s
300 s
360 s
420 s
PSS
Ti
Matrigel
Col I
B
APTES
RGD
PPAAm
PEI
C
PPI-G4
PDADMA
40 µm

Supplementary Figure S5. Representative example of the intracellular Ca^{2+} mobilization in vital fluo-3 loaded MG-63s growing on (A) negatively, (B) moderately positively and (C) highly positively charged surfaces in the time frame 0-420 s. First, the basal Ca^{2+} signal is recorded, and then the cells are stimulated with ATP at 180 s (marked by the red box). Note the impressively higher Ca^{2+} signals after ATP stimulation in cells on moderately positively charged surfaces (240 and 300s). Ca^{2+} mobilization in cells on highly positively charged surfaces is strongly impaired, accompanied by a spherical cell morphology and reduced cell area (magnification 40x, scale bars 40 µm; LSM780).

Supplementary Table S2. Chemical composition of modified Ti by XPS (mean ± SD, n = 3).

**Surface**		**Ti**	**Si**	**C**	**O**	**N**	**Other**
**Control**	PPAAm	0.0	0.0	74.2 ± 0.4	1.6 ± 0.3	24.3 ± 0.1	< 4 at-%
**Amino Polymers**	APTES	8.1 ± 0.5	6.4 ± 0.1	40.9 ± 1.0	37.5 ± 0.6	6.4 ± 0.1	< 4 at-%
	PEI	16.8 ± 0.1	0.0	29.2 ± 0.5	49.3 ± 0.2	4.7 ± 0.8	< 4 at-%
	PPI-G4	4.0 ± 2.5	1.5 ± 0.5	57.7 ± 3.4	23.7 ± 2.5	13.3 ± 1.4	< 4 at-%
**ECM & Peptides**	Col I	0.0	0.0	55.9 ± 1.4	25.8 ± 1.5	11.3 ± 1.1	7.1 at-%*
	Matrigel	27.4 ± 0.6	0.0	16.2 ± 0.6	53.3 ± 0.4	2.1 ± 0.3	< 4 at-%
	RGD	7.6 ± 0.1	4.9 ± 0.2	40.6 ± 0.3	37.7 ± 0.3	7.0 ± 0.3	< 4 at-%
**Polyelectrolyte Multilayers**	PSS	0.0	12.5 ± 0.5	51.8 ± 2.1	19.8 ± 0.0	4.8 ± 0.1	11.2 at-%*
	PDADMA	0.0	0.1 ± 0.0	77.9 ± 0.2	13.0 ± 0.2	5.2 ± 0.0	< 4 at-%

* Col I: mainly 4.5 ± 0.7 at-% Na, 1.6 ± 0.3 at-% S; PSS: mainly 4.3 ± 2.0 at-% Na, 5.4 ± 0.2 at-% S.

Supplementary Methods

Relative cell viability

As cells on the highly positively charged surfaces PPI-G4 and PDADMA exhibited altered morphology and disturbed Ca^{2+} mobilization after 24 h, the relative cell viability of MG-63s on these surfaces was determined by MTS assay in comparison with the control surfaces Ti and PPAAm. This test utilizes the ability of NADH(P)H-dependent dehydrogenase enzymes in mitochondrial active and viable cells to reduce yellow MTS ((3-(4,5-dimethylthiazol-2-yl)-5-(3-carboxymethoxyphenyl)-2-(4-sulfophenyl)-2H-tetrazolium salt) to purple formazan. A detailed description of the procedure can be obtained from Staehlke et al. 2018. In brief, 50,000 cells/cm^2 were seeded onto the surfaces for 24 h and then incubated in a fresh 24-well plate for 2–3 h in 500 µl DMEM containing 100 µl of MTS reagent (CellTiter 96 ® Aqueous ONE-Solution Cell Proliferation Assay, Promega, USA) at 37 °C. The increase in color intensity in the media was quantified by measuring the absorbance of the supernatants in a 96-well plate at 490 nm with a microplate reader (Anthos, Mikrosysteme, Krefeld, Germany). A background measurement was recorded at 650 nm to be subtracted from the absorption values. Cell numbers were determined by crystal violet staining. Therefore, cells were fixed by shaking with methanol (J. T. Baker, Deventer, Netherlands) for 10 min at RT. The fixing solution was discarded, the cells covered with a Neißer solution (Carl Roth, Karlsruhe, Germany) and incubated for 10 min shaking at RT. Distilled water was then added and aspirated until the supernatant was clear. The crystal violet was re-dissolved by incubating the cells with 33% acetic acid (J. T. Baker, Deventer, Netherlands) for 10 min shaking at RT. The optical density of the transferred supernatants was quantified at 620 nm. The relative cell viability was calculated by the quotient of MTS and crystal violet values and normalized to the Ti control. Three independent experiments were carried out, each in duplicate (n = 3).

Cell Proliferation

To investigate the cell proliferation, 80,000 cells were seeded onto Ti, PPAAm, PPI-G4 and PDADMA surfaces for 24 h and then trypsinated with 0.05% trypsin/0.02% EDTA (PAA Laboratories, Pasching, Austria) for 7 min. After centrifugation (10 min, 1200 rpm; Centrifuge 5702 R, Eppendorf AG, Hamburg, Germany) cells were fixed with 70% ethanol at -20 °C overnight. Subsequently, cells were washed once with PBS, treated with RNase (1 mg/ml, Sigma-Aldrich Chemie, Taufkirchen, Germany) at 37 °C for 25 min and incubated with propidium iodide (PI, 50 mg/ml, Sigma-Aldrich Chemie, Taufkirchen, Germany) at 4 °C overnight. Up to 10,000 events per sample were measured with the flow cytometer FACSCalibur (BD Biosciences, San Jose, CA, USA) using the software CellQuest Pro 4.0.1 (BD Biosciences). For the cell proliferation analysis the cell cycle phases $G1_{sub}$, G0/G1, S and G2/M were calculated in percent using FlowJo v10 (BD Biosciences, San Jose, CA, USA). In accordance with Staehlke et al. 2019, we defined the MG-63s in S- and G2/M-phase as proliferative cells and in the sub-G1 phase as apoptotic cells for statistical evaluation (n = 3 independent experiments).

Cell viability for ATP concentration

To ensure that an adequate amount of adenosine 5`-triphosphate (ATP) was used to stimulate the intracellular calcium (Ca^{2+}) mobilization in MG-63 osteoblasts, an MTS assay was performed. For this purpose, 10,000 cells/well were seeded into 96-well plates (Greiner Bio-One, Frickenhausen, Germany) and cultivated for 24 h. The culture medium was then removed, and the cells incubated for 20 minutes with ATP concentrations of 0 (as control), 0.5, 1, 5 and 10 mM in isotonic HEPES (4-(2-hydroxyethyl)-1-piperazineethanesulfonic acid) buffer, as used for Ca^{2+} mobilization. After the

addition of ATP, the cell morphology was observed with a light microscope (Axiovert 40 C, CP-Achromat 10x/0.25 Ph1, Carl Zeiss, Jena, Germany) and an eventual pH change in the HEPES buffer determined with a MicroFET pH-meter (Sentron Europe BV, Leek, The Netherlands). Thereafter, the HEPES buffer was replaced with 100 µl culture media containing 20 µl of MTS reagent (CellTiter 96 ® Aqueous ONE-Solution Cell Proliferation Assay, Promega, USA) and the cells were incubated for 1 h at 37 °C. The absorbance of the supernatants in a 96-well plate was measured with a microplate reader (Anthos, Mikrosysteme, Krefeld, Germany) at 490 nm. A background measurement was recorded at 650 nm to be subtracted from the absorption values. Three independent experiments were carried out, each in triplicate (n = 3).

ATP receptor immunofluorescence staining

MG-63 cells (30,000 cells/cm²) were cultured on the Ti arrays for 24 h and then fixed with 4% PFA (Sigma-Aldrich Chemie, Taufkirchen, Germany) at RT for 10 min. After washing three times in PBS, cells were permeabilized with 0.1% Triton X-100 (10 min, RT) (Merck, Darmstadt, Germany). ATP receptor staining was performed using the anti-$P2Y_2$ antibody (1:100, Alomone Labs, Jerusalem, Israel, APR-10) and the anti-$P2X_7$ antibody (1:200, Alomone Labs, Jerusalem, Israel, APR-04). Cells were incubated with the primary antibody for 60 min at RT. After washing with PBS, the cells were treated with goat-anti-rabbit-IgG-Alexa Fluor 488 (1:200, Invitrogen, Karlsruhe, Germany, A-11008) for 30 min at RT in the dark. Afterwards, samples were embedded with DAPI containing Fluoroshield™ on a cover slip and stored in the dark at 4 °C. The ATP receptors were examined using the LSM780 (Carl Zeiss) with a Plan-Apochromat 63x/1.40 Oil DIC M27 objective (Carl Zeiss) and the ZEN software (ZEISS efficient navigation, ZEN 2011 SP4, black edition, Carl Zeiss).

Intracellular Ca^{2+} mobilization

Pilot test: In order to determine the origin of the Ca^{2+} ions for their cytoplasmic increase in the cells, more general testing was carried out first. Ca^{2+} ions either originate e.g. from the intracellular Ca^{2+} storage – the endoplasmic reticulum (ER), which releases Ca^{2+} into the cytoplasm after activation of the phospholipase-C signaling pathway – or they originate from extracellular sources and are transported into the cell via voltage-sensitive Ca^{2+} channels/ ligand-gated ion channels, or a mixture of both. For this purpose, extracellular Ca^{2+} sources had to be eliminated from the HEPES buffer. The HEPES buffer composition including 1 mM $CaCl_2$ was used, as previously published (Staehlke et al., 2015, 2018), as well as a HEPES buffer without $CaCl_2$. Washing and staining steps (fluo-3) were performed without $CaCl_2$ in PBS and HEPES buffer. The following recording of Ca^{2+} signal and analysis of Ca^{2+} time course was performed as described in the materials and methods section of the manuscript.

Staehlke, S., Koertge, A., and Nebe, B. (2015). Intracellular calcium dynamics dependent on defined microtopographical features of titanium. *Biomaterials* 46, 48–57. doi:10.1016/J.BIOMATERIALS.2014.12.016.

Staehlke, S., Rebl, H., Finke, B., Mueller, P., Gruening, M., and Nebe, J. B. (2018). Enhanced calcium ion mobilization in osteoblasts on amino group containing plasma polymer nanolayer. *Cell Biosci.* 8, 22. doi:10.1186/s13578-018-0220-8.

Staehlke, S., Lehnfeld, J., Schneider A., Nebe J. B., Müller R. Terminal chemical functions of polyamidoamine dendrimer surfaces and its impact on bone cell growth. *Mater. Sci. Eng. C* 101, 190–203. doi: 10.1016/j.msec.2019.03.073
